# Grouping compositions based on similarity of music themes

**DOI:** 10.1371/journal.pone.0240443

**Published:** 2020-10-08

**Authors:** Barbara Laskowska, Mariusz Kamola

**Affiliations:** NASK National Research Institute, Warsaw, Poland; University of Sao Paulo, BRAZIL

## Abstract

Finding music pieces whose similarity is explainable in plain musical terms can be of considerable value in many applications. We propose a composition grouping method based on musicological approach. The underlying idea is to compare music notation to natural language. In music notation, a musical theme corresponds to a word. The more similar motives we find in two musical pieces, the higher is their overall similarity score. We develop the definition of a motive as well as the way to compare motives and whole compositions. To verify our framework we conduct a number of grouping and classification experiments for typical musical corpora. They include works by classical composers and examples of folk music. Obtained results are encouraging; the method is able to find non-obvious similarities, yet its operation remains explicable on the ground of music history. The proposed approach can be used in music recommendation and anti-plagiarism systems. Due to the musicological flavor, one of potentially best applications of our method would be that in computer assisted music analysis tools.

## 1 Introduction

Music and computer technologies specialists have been cooperating for a long time especially in the area of music analysis. Despite that a lot of work has been done, musicologists are not convinced about algorithmic capabilities to support analytical work on musical pieces. The abstract nature of musical language requires a context-aware approach to music analysis, which is difficult to present in machine language [1].

As the research advances, further questions arise about what approach is the most appropriate. While the composition itself is written down formally, an important aspect of understanding music is its emotional load. That is why musicologists are more interested in some specific application than just a computer representation of music [2]. For different goals, one should process music differently. One can find a number of scientific experiments showing that people focus on different elements of music depending in the task they have to to do [3]. It means that music analysis can be very contextual. Lerdahl and Jackendoff [4] introduce musical grammar based on linguistic grammar, which allows to organize music logically. However, the beauty of music lies in the non-obvious solutions that do not always obey logical rules.

Our aim is to group musical works in a way similar to methods used for grouping text documents, which are based on words analysis. An element of music that can be understood like a musical word is called a motive—a few selected consecutive notes that carry some musical meaning. Musical pieces can be compared for similar or identical music themes. We want to develop a framework for detection of meaningful motives in compositions, and for computation of their degree of similarity.

Our main research problem is how to define a motive so that it can be compared to motives in other compositions, yet its structure will account for music properties musicians commonly find essential in terms of similarity. This study is therefore aiming for an analogue method of traditional musical analysis, as it applies a weighing scheme to melody properties. Such idea is something that can convince musicologists about practicality of computer music analysis because they can easily interpret obtained results.

## 2 Related work

Musical language has abstract nature, therefore its formalization is difficult. There are lots of possibilities of working with musical pieces, and various approaches to the processing of musical materials. Some scientists process raw audio, others work with static music notation.

Audio signal processing requires the use of transforms and other tools but allows the analysis of a specific interpretation that carries information related to emotional charge [5]. Performers often use small changes in the tempo of performance of the composition, a short stop at important moments, which are not always written directly in the notes. However, the analysis of a musical work on the basis of its description is more formal and allows the interpretation of the work as it was composed instead of the performers’ interpretation. Principles are based on musicologists’ approach so they are easily understandable by them. Our aim is to analyze compositions rather than particular interpretations. Hence in the following sections 2.1 and 2.2 we present an overview of work closely related to our approach, i.e. the way motives get defined and, subsequently, used in overall musical piece similarity computation.

### 2.1 Motives identification

The fundamental idea of our work is to analyse a musical piece similarly to how text documents are processed. In natural language, a word constitutes a basic element of the message. In musical language, a word would correspond to a motive. Unfortunately, the definition of motive is not fixed. Musical motive is commonly understood to be a melodic, harmonic or rhythmic pattern of any length [6].

A number of attempts have been made over the years to identify motives in various ways, where the rhythm, melody, harmony and contour are considered the relevant musical properties. However, researchers differ in perception of their importance: Hugo Riemann originally emphasized rhythmic data [7], while others later on valued pitch intervals [8]. All musicologists agree that the universal motive feature is its repetition in a music piece. Longer motives appear naturally less often than short ones; therefore finding a criterion for motive length boundary is important. While typical motive length is genre specific, it has been observed that note duration tends to be shorter at the beginning of a musical phrase [9]. Cenkerová utilizes the phenomenon and proposes a segmenting model based on non-descending note duration [10]. Wilder [1] identifies motives by searching for potential boundaries of segmentation, determined by some threshold for so called delta functions. Delta functions are defined over melodic, contour and rhythmic properties, and a sequence of notes is considered as a motive if both delta function values and number of occurrences are satisfactory. Cambouropoulos [11] also combines contour and rhythmic properties; he claims that the earlier ones can be represented just by indicators of subsequent notes diatonic pitch (zero or non-zero, with direction info), and the latter ones by duration ratios. If considered jointly, they provide good pattern search space. Similarity metrics is defined for a sequence of *n* notes, and used to extract motives; *n* being a parameter.

The paradigm that a motive is a repeated entity poses problem of efficient representation of its constituting patterns with support for their overlapping and cyclicity. Lartillot [12] proposes to use pattern occurrences trees for this purpose. Pattern trees resemble suffix trees, applied widely in various natural language processing tasks [13]. Pattern trees used in [12] make it possible to work with more and less specific description of motives.

The work reported so far was done for monodic music pieces. Addressing polyphony moves us even further away from natural language analogies, yet it is inherent in music and was addressed in the research. Meredith *et al*. [14] develop four algorithms that consider polyphonic music data sets. They search for maximal repeated point sets in Euclidean space. Such geometric approach allows to effectively find patterns that differ by added or removed notes, but does not take into account changes such as augmentation, dilatation or reflection. It also does not account for small interval or rhythmic changes.

### 2.2 Similarity index

Someone’s decision about a method of calculating music similarity is something of arbitrary. There exist three general approaches, differing firstly by how a music piece is represented [15]. The *sequential* one represents a piece by a set or a sequence of elements (points, sets, tuples etc.). The *structural* one develops a tree-like or graph structure of elements that additionally accounts for relations between elements (inclusion, cyclicity etc.). Finally, the *abstracted* approach applies typical distance measures, but pieces are represented by points in a developed space of their salient but abstract features.

Finding similarity of sequences usually involves defining an edit distance function that will account for differences in compared sequences. Many studies employ Levenshtein distance for this purpose, either in its original form or adapted. Levenshtein distance counts edit operations (insertion, deletion and change) needed to transform one sequence into another. For instance, Frieler *et al*. [16] apply the method to rate similarity of phrases defined as sequences of pitch and duration info, and similarity value is the complement of reversed Levenshtein distance, normalized to the longer of the two sequences being compared. The authors use arbitrary values for similarity thresholds, based on expert insight. Rolland x [17] uses a ranked list of matches found by a content-based music retrieval system in response to a short musical query about a whistled or hummed melody. System transforms melody to MIDI file and compares it against music database. Pairings of note sequences are created and put in correspondence called alignment. The value of alignment is calculated as a sum of relevant pairings contribution. The greatest value of all possible alignments is considered the similarity.

If adapted skillfully, edit distance calculation can be accomplished rapidly, thus facilitating efficient database queries. Weyde and Datzko [18] encode rhythm and pitch contour (going down, going up, or equal) for fixed pattern of nine notes in order to pack it into a 32-bit integer. Edit distance can be calculated very fast in such setting. Additionally, frequency statistics are calculated for such “9-note words” for the whole musical corpus, to allow weighing of similar patterns. Rare patterns get more weight, exactly as in TF-IDF (term frequency—inverse document frequency) weighing scheme, which is a standard method in text document retrieval tasks. Pieces that contain similar patterns, appearing with similar frequencies, are deemed to be similar in general.

Structural approaches to music comparison are rooted in musical grammar rules, laid out in [4], which allow to evaluate structural importance of neighbouring notes or note sequences. Recursively, one can construct a tree for a music piece, and develop similarity measures for such trees. For instance, Matsubara *et al*. [19] propose a distance measure based on earlier studies, that combines pitch and duration information. The idea is to calculate edit distance based on tree properties of notes—especially the total of time span of notes in the underlying sub-trees.

The abstracted approach is accomplished by machine learning methods, which come in many types and have proven to be successful in classification tasks in other domains. Parameter space can be constructed in many ways: Müllensiefen and Wiggins [20] propose pitch contour to be approximated by a multinomial function of some predefined order. The obtained parameter space is technically understandable, but carries little meaning for a musicologist. Yet less explainable are so called embeddings, i.e. vectors of input data features that capture its context—that is, co-appearance with other bits of data. In the realm of natural language processing, the term *word2vec* has been coined, since separate words get translated into vectors, and selected relations of word meanings, as similarity or analogy, can be represented by simple geometric algebra. Alvarez and Gómez-Martin [21] follow such approach to build embeddings for music fragments (either pitch or duration contour) in order to compare folk songs by calculation of cosines between their key embeddings.

### 2.3 Classification of music

There are several attempts at computer classification of music. Most of them are based on statistical metrics like harmonic or melodic interval, harmonic and melodic *n*-grams, pitch duration, etc. Approaches to classification depend on particular classification task.

Manaris *et al*. [22] perform experiments with artificial neural networks. They test possibilities of applying Zipf’s law to music classification, i.e., power law distribution of occurrences of music patterns. The experiments address three criteria: author attribution, style identification and “pleasantness” prediction. The authors claim that “pleasantness” measure reflects music aesthetics best. The neural networks reached success rate of about 95%.

Another study [23] tested five different classification models: decision trees, logistic regression, rule base, naive Bayes and SVM, to tell apart compositions by Bach, Haydn and Beethoven. Classification accuracy rated from 80 to 86%, which can be considered a success. However, while the models used are relatively transparent for a skilled data scientist, they present themselves quite opaque for a musicologist. It is because they use advanced algebra concepts in combination with high-dimensional vector of music piece statistics, incompatible with practice used in studies in music theory or history. The disadvantage of such classification is therefore the difficulty in obtaining valuable musicological interpretation of its results.

## 3 Proposed approach

An apparent analogy between a musical piece and a text document has become the initial inspiration for our study. Music theory divides the melody for elements such as period, sentence, phrase, motive, which consist of notes and rests [4]. In natural language, documents are compared by significant text tokens, e.g., words they contain. Techniques of natural language processing have been successfully used in other studies [18, 21]. In our work, we decided to focus on the definition of significant motives, which are then used to compare musical pieces. A motive is a short musical figure that carries melodic meaning, so it naturally corresponds to a word in a text. It affects the way the listener receives music. Motives can characterize a given composition, but also the composer’s style.

**Fig 1:**
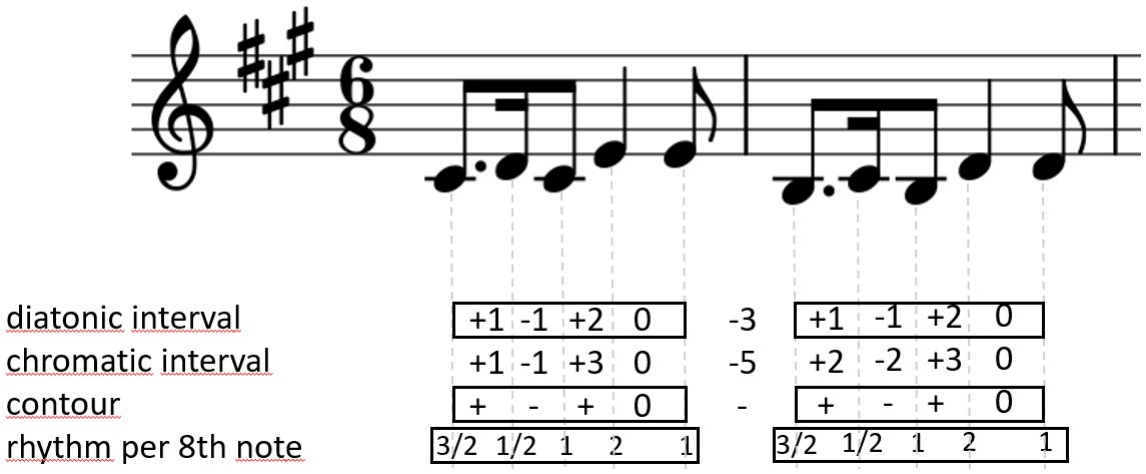
A short melody with description in four dimensions. Recurring value sequences, which are realisations of a motive, have been put in frames.

To make a formal definition of motive that would make it possible to compare motives clearly and comprehensibly is a challenging task. Two basic music elements that affect human music perception and allow to recognize motives, compositions or a composer, are melody and rhythm. We decided to consider both two elements in our definition of a motive, like in other studies [11, 16, 18]. Lartillot points out as many as four main parametric dimensions that sufficiently describe motives [12]. These are: chromatic interval, diatonic interval, contour and rhythmic value. An example of a melody fragment described with these dimensions is shown in Fig 1. Numbers or signs located between pairs of notes stand for differences between the notes in each dimension. Numbers located at notes describe properties of the notes themselves.

Another difficulty associated with the definition of a motive is that a given motive may get instantiated in different ways throughout a composition. Therefore, we can recognize a motive as a frequently repeated short musical fragment, and we have to account for its different versions.

To formalize the above spontaneous description of what a motive is, let us start with defining a music work, or a composition *W*_*i*_, as a sequence of sounds

$$\begin{array}{c} W_{i}=(\left(\pi_{1},\delta_{1}\right),\left(\pi_{2},\delta_{2}\right),\ldots )\;, \end{array}$$ (1)

where pairs (*π*, *δ*) stand for pitch and duration of subsequent notes or rests. Model (1) is appropriate for works without co-occurring notes, let alone chords or individual melodic lines. Here we work only with such compositions, much like most of mentioned other studies.

Since music elements are not strict counterparts of written language constructs, we cannot rely on word-based analysis, so popular and useful in computational linguistics. But we can still employ *n*-gram approach, already reported in [24]. In text processing, *n*-grams are subsequent sequences of letters of fixed length *n*, e.g. word “science” can be decomposed into 3-grams: “sci”, “cie”, “ien”, “enc”, “nce”. Substring length *n* is a global parameter for modeling. Likewise, let a musical *n*-gram *Γ* be a sequence

$$\begin{array}{c} \Gamma_{j}=(\left(\pi_{j},\delta_{j}\right),\ldots ,\left(\pi_{j+n-1},\delta_{j+n-1}\right))\;. \end{array}$$ (2)

Consequently, *n*-gram representation of *W*_*i*_ can be written as

$$\begin{array}{c} G_{i}=(\Gamma_{1},\Gamma_{2},\ldots )\;. \end{array}$$ (3)

and the corresponding set of *unique* *n*-grams as $G_{i}^{⋆}$.

Following the proposition by Lartillot [12], we can extract from every musical *n*-gram in $G_{i}^{⋆}$ its relevant features, i.e. chromatic intervals, diatonic intervals, contour and rhythmic values, in form of a 5-tuple of vectors

$$\begin{array}{c} R_{j}=F\left(\Gamma_{j}\right)=(\text{a},\text{b},\text{c},\text{d},\text{e})\;, \end{array}$$ (4)

where the transformation F computes

a vector of chromatic intervals between subsequent notes, $\text{a}\in Z^{n-1}$ (integer numbers);
b vector of diatonic intervals between subsequent notes, $\text{b}\in Z^{n-1}$;
c vector of contour (pitch change direction between subsequent notes), **c** ∈ {−1, 0, 1}^*n*−1^;
d vector of subsequent notes duration, $\text{d}\in Q^{n}$ (rational numbers).
e vector of ratios of subsequent notes duration, $e_{i}=\frac{d_{i}}{d_{i+1}},\;\text{e}\in Q^{n-1}$.

We call *R*_*j*_ here a *fingerprint* of *n*-gram Γ_*j*_ just because it represents those musical properties we consider characteristic for a music piece. Feature vector **e** is rather superfluous, but we prefer to use it further instead of the original vector **d**, proposed in [12]. The idea of characteristic features extraction, termed fingerprinting, has originally started with physical devices, like typewriters [25], and gradually was extended in order to distinguish non-physical entities, like web browsers [26]. We use it to distinguish *n*-grams and whole music pieces.

We define a musical motive *M*_*ix*_ present in *W*_*i*_ as a set of similar fingerprints, called *realizations* of that motive. Therefore, we follow the sequential modeling approach, yet the novelty is to add capability to consider slightly differing fingerprints as one motive—much like in the structural approach. Because there can exist a number of motives in a music work, we enumerate them and get *x*-th motive present in *W*_*i*_ defined with use of all indexes and parameters as a set:

$$\begin{array}{c} M_{ix}^{\left(n\right)}=\{R_{j},\;j\in \Omega_{ix}^{\left(n\right)}\}\;, \end{array}$$ (5)

where $\Omega_{ix}^{\left(n\right)}$ is a set indexing *n*-gram based realizations, defined as

$$\begin{array}{c} \Omega_{ix}^{\left(n\right)}=\{k:\exists l\in \Omega_{ix}^{\left(n\right)}\wedge k\neq l\wedge \sigma \left(R_{k},R_{l}\right)\geq \theta \}\;. \end{array}$$ (6)

That is, any realization of a motive must be similar with strength *θ* or more to at least one of other realizations of the same motive. Similarity of realizations is defined by function *σ*(⋅), which is based on modified Hamming distance, with weights depending on **a**, **b**, **c** and **e**. This way, we adapt the weighing technique that was used by others to determine motive boundary [1] or to compare motives [16], for the purpose of the very motive creation.

We constructed the weighing scheme expertly to take into account modifications of motives which are frequently applied in musical practice. Examples of such modifications will be presented later in Fig 3. Function *σ*(⋅) performs pairwise computation of two components, over subsequent elements of fingerprint vectors. Component *μ*(⋅) handles melody similarity, while component *α*(⋅) processes the rhythm:

$$\begin{array}{c} \sigma \left(R_{k},R_{l}\right)=\frac{1}{n-1}\sum_{i\in 1\ldots n-1}[\mu \left(a_{ki},a_{li},b_{ki},b_{li},c_{ki},c_{li}\right)+\alpha \left(e_{ki},e_{li}\right)]\;, \end{array}$$ (7)

**Fig 2:**
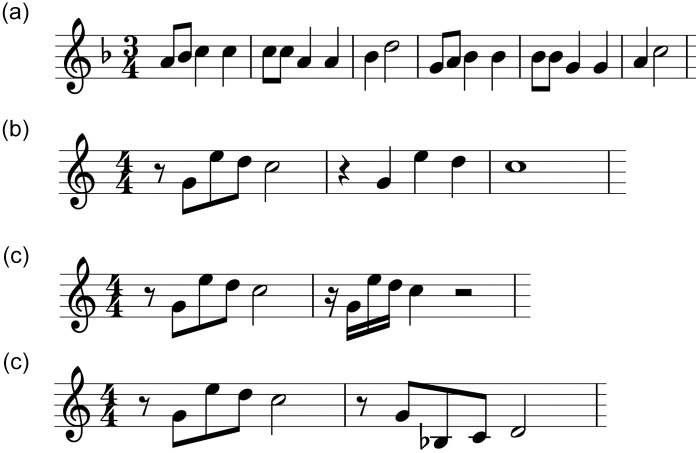
Common types of modification of motives. (a) Transposition: sequence of notes is moved up or down by a constant interval. (b) Augmentation: lengths of notes are prolonged in other realizations. (c) Diminution: lengths of notes are shortened in other realizations. (d) Reversal: intervals occur in the opposite direction.

Vectors **a** and **b** represent diatonic and chromatic intervals. If two chromatic intervals of two notes sequences are identical then usually the diatonic intervals are also identical. But if diatonic intervals are identical it is not very common that chromatic intervals are identical, too (for example in diatonic transposition, cf. Fig 2a; the importance of contour in recognizing transpositions has been reported in [27], here we address it differently). Either of the types of intervals is musically meaningful, but the fact that both are identical at the same time does not strengthen the evidence of the higher overall similarity of the works. Therefore, *μ*(⋅) value will be calculated elementwise from either **a**_*k*_ vs. **a**_*l*_ or **b**_*k*_ vs. **b**_*l*_ comparisons, whichever holds true.

Equality of vectors **c**_*k*_, **c**_*l*_ has additional influence on similarity, but the contour itself is less important than the interval value. Imitation techniques as reversal, cf. Fig 2d, do not preserve contour values but are perceived effectively as almost exact repetitions of the motive. This fact is reflected by the below detailed formulas for similarity. The rhythmic component is simpler; it depends only on the ratios between subsequent notes duration. Therefore, we score similarity for imitation techniques such as augmentation and diminution (Fig 2b and 2c) as follows:

$$\begin{array}{c} \mu \left(a_{ki},a_{li},b_{ki},b_{li},c_{ki},c_{li}\right)=\{\begin{array}{cccc} 1 & \text{if} & \left(a_{ki}=a_{li}\vee b_{ki}=b_{li}\right)\wedge c_{ki}=c_{li} & ,\text{else}\\ \frac{1}{2} & \text{if} & \left(a_{ki}=a_{li}\vee b_{ki}=b_{li}\right)\wedge \neg \left(c_{ki}=c_{li}\right) & ,\text{else}\\ \frac{1}{3} & \text{if} & \neg \left(a_{ki}=a_{li}\vee b_{ki}=b_{li}\right)\wedge c_{ki}=c_{li} & ,\text{else}\\ 0 & & \end{array}\;, \end{array}$$ (8)

$$\begin{array}{c} \alpha \left(e_{ki},e_{li}\right)=\{\begin{array}{cc} 1 & e_{ki}=e_{li}\\ 0 & \text{else} \end{array}\;. \end{array}$$ (9)

The way motives get defined (5), (6) results in that a single motive consists of similar fingerprints, which usually means that motives extracted from one music piece are formed of disjoint sets of fingerprints. This is fine because we want a motive to stand for distinct features of a music piece. In order to compute similarity of two given motives, we propose Jaccard distance

$$\begin{array}{c} \zeta \left(M_{x},M_{y}\right)=\frac{|M_{x}\cap M_{y}|}{|M_{x}\cup M_{y}|} \end{array}$$ (10)

with piecewise equality relation for motive fingerprints. Jaccard distance does not require setting of any threshold-like parameter, and is conveniently normalized to interval 〈0, 1〉.

So far, we considered a motive be based on *n*-grams, with *n* fixed—hence the upper index in (5). The definition can be extended easily for multiple values of *n*; in such case the motive is just a sum, $M_{ix}=\cup_{k}M_{ix}^{\left(n_{k}\right)}$. In our experiments we composed motives with *n*-grams for *n* running from two to seven.

The formulation of music works similarity cannot be just a straightforward extension of formula (10). In practice, works get recognized by a listener as similar from their elements that match best. Therefore, we propose similarity of two works to be the normalized sum of similarities of distinct pairs of motives, when best matched:

$$\begin{array}{c} s\left(W_{i},W_{j}\right)=\underset{V}{\text{max}}\frac{\sum_{x}\sum_{y}v_{xy}\zeta \left(M_{ix},M_{jy}\right)}{\sum_{x}\sum_{y}v_{xy}}\;. \end{array}$$ (11)

In (11), **V** stands for binary match matrix with number of rows equal to the number of motives in *W*_*i*_, and with number of columns equal to the number of motives in *W*_*j*_. Consequently, constraints on **V** get defined as follows

$$\begin{array}{c} \forall_{x}\sum_{y}v_{xy}\leq 1\;,\;\forall_{y}\sum_{x}v_{xy}\leq 1\;,\;v_{xy}\in \left\{0,1\right\}\;, \end{array}$$ (12)

i.e., there can be at most one match for each *W*_*i*_ motive (constraint on row sums) and at most one match for each *W*_*j*_ motive (constraint on column sums).

We could not directly transfer algorithms from text document classification methods to the field of music due to complex definition of a motive—especially, we had to discard those algorithms based on concept of cosine similarity of works. Vectors in parameter space would be too far apart, or the space would be too big for effective association of important correlations between sequences of melodies, unlike for motive embeddings of size 150, used in [21]. Instead, we used agglomerative clustering, with distance defined as the inverse of similarity (11). Adjustment of the grouping cutoff value, or cophenetic distance, allowed examination of variants with different number of output classes.

Grouping correctness was verified in two ways. The objective metrics is a kind of *precision* defined over classes that prevail in the output groups:

$$\begin{array}{c} q=\frac{\sum_{i}^{N}H_{i}^{⋆}}{\sum_{i}^{N}H_{i}}\;. \end{array}$$ (13)

Here, *H*_*i*_ is the number of works in output group *i*, while $H_{i}^{⋆}$ is the maximal number of works belonging to the same input class that can be found in the output group *i*. Precision defined this way is related inversely to the concept of entropy, as input classes that get separated into different groups result in higher score. At the same time, it resembles typical precision definition in classification tasks, but with support of non-square confusion matrix.

The subjective approach is by expert assessment of music content of pieces that have been grouped differently, and deduction of the underlying reasons.

## 4 Results

We conducted a series of experiments to verify our approach. We analysed two collections of musical compositions. The first set consists of classical composers’ pieces: Bach, Monteverdi and fourteenth century Italian composers. The second one is a collection of folk music songs. Both datasets are provided as, S1 and S2 Datasets, respectively. We analysed pieces grouped with our approach w.r.t. original piece classification in the corpus, and alternatively, the type of composition. We also looked in detail at cases that deviate from obvious and expected classifications. This means we checked if compositions grouped together by our model are indeed similar in terms of motives content.

### 4.1 Motives construction

**Fig 3:**
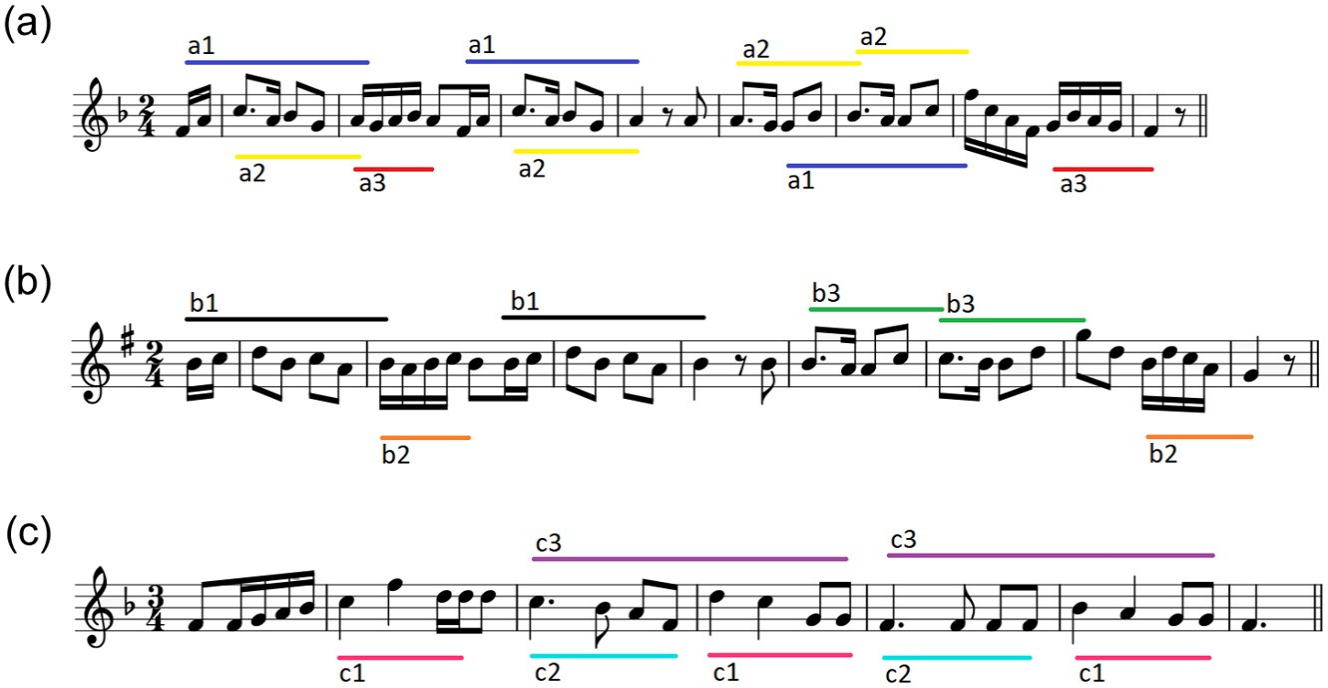
Examples of three short folk songs that have been grouped together. Some motives have been identified in the figure. Realizations of the same motive were marked with the same names, e.g. “a3”. Songs (a) and (b) have high similarity. It is probably the same melody that has changed through various modifications of key, melody and rhythm in oral transmission. Song (c) is distinctly different.

Fig 3 shows an example of a small output group of size three of folk music clustering. Similarity of melodies Fig 3a and 3b is one of the highest in this experiment. Ethnomusicological analysis of these short fragments leads to a conclusion that we have found two versions of the same melody. It is common in folk music that songs had changed trough oral transmission in various modification of keys, melody and rhythm before melodies were written down.

Similarity of composition Fig 3c to the previous two ones is low. It differs in time signatures and melody division. In compositions Fig 3a and 3b we have four-bar antecedent and consequent, while composition Fig 3c consists of three two-bar elements that look like introduction, and then antecedent and consequent phrases. What we can take for similarity is for example notes duration. In its first part, the melody is built on shorter notes than in parts that follow. This also holds for compositions Fig 3a and 3b but is not so clear as in Fig 3c. Compositions Fig 3a and 3c share the same key and they begin and end with the same notes and chords. But we cannot easily see the similarity based on musical themes. Motives a2 and b3 in compositions Fig 3a and 3b are the only ones unquestionably similar to motive c2 in Fig 3c. Altogether, the two first melodies are not very similar to the last one, yet the nature of this similarity can be interesting from musicological point of view.

We show how the calculations of motive realizations similarity (7) is done, taking an example of motives a2 and b3. Fingerprints of realizations, in order of appearance, are provided in Table 1. We use function *σ* to calculate similarities and to group realisations into sets that represent motives. Below we present only a few exemplary calculations, first, for motive a2:

$$\begin{array}{c} \sigma \left(R_{1},R_{2}\right)=\left(\underset{\mu }{\underset{︸}{\frac{1}{4}\cdot 1+\frac{1}{4}\cdot 1+\frac{1}{4}\cdot 1+\frac{1}{4}\cdot 1}}\right)+\left(\underset{\alpha }{\underset{︸}{\frac{1}{4}\cdot 1+\frac{1}{4}\cdot 1+\frac{1}{4}\cdot 1+\frac{1}{4}\cdot 0}}\right)=1.75 \end{array}$$ (14)

$$\begin{array}{c} \sigma \left(R_{3},R_{4}\right)=\left(\frac{3}{4}\cdot 1+\frac{1}{4}\cdot 0\right)+\left(\frac{3}{4}\cdot 1+\frac{1}{4}\cdot 0\right)=1.5 \end{array}$$ (15)

$$\begin{array}{c} \sigma \left(R_{2},R_{4}\right)=\left(\frac{1}{4}\cdot \frac{1}{3}+\frac{1}{4}\cdot 0+\frac{1}{4}\cdot \frac{1}{2}+\frac{1}{4}\cdot \frac{1}{3}\right)+\left(\frac{3}{4}\cdot 1+\frac{1}{4}\cdot 0\right)\approx 1.04 \end{array}$$ (16)

The similarity between motive b3 realizations is:

$$\begin{array}{c} \sigma \left(R_{5},R_{6}\right)=\left(\frac{3}{4}\cdot 1+\frac{1}{4}\cdot 0\right)+\left(\frac{3}{4}\cdot 1+\frac{1}{4}\cdot 0\right)=1.5 \end{array}$$ (17)

Finally, we can calculate the overall similarity of the two motives by counting identical realizations:

$$\begin{array}{c} \zeta \left(M_{a2},M_{b3}\right)=\frac{|M_{a2}\cap M_{b3}|}{|M_{a2}\cup M_{b3}|}=\frac{\left|\{R_{3}\equiv R_{5}\}\right|}{\left|\{R_{1}\equiv R_{2},R_{3}\equiv R_{5},R_{4},R_{6}\}\right|}=\frac{1}{4} \end{array}$$ (18)

When we have all motives representing compositions from Fig 3, we can find the best pairings based on the similarity measure between motives. The mean of motives similarity for the best match is the similarity of compositions.

**Table 1:** Numerical representation of motives a2 and b3 from compositions in Fig 3.

finger-print	Motive a2																Motive b3							
	*R*_1_				*R*_2_				*R*_3_				*R*_4_				*R*_5_				*R*_6_			
**a** diatonic	3	2	3	2	3	2	3	2	2	1	3	1	2	1	3	4	2	1	3	1	2	1	3	4
**b** chromatic	3	1	3	2	3	1	3	2	2	0	3	0	1	0	3	5	2	0	3	0	1	0	3	5
**c** contour	-	+	-	+	-	+	-	+	-	0	+	0	-	0	+	+	-	0	+	0	-	0	+	+
**e** rhythm	3	1/2	1	2	3	1/2	1	1/2	3	1/2	1	2/3	3	1/2	1	2	3	1/2	1	2/3	3	1/2	1	1

### 4.2 Music piece classification experiment

To confront our approach with a priori classification, we carried out hierarchical clustering of a balanced set of 90 music pieces, attributed originally to three classes: “Bach”, “Monteverdi” and “Trecento”. The pieces had been picked randomly from public Music21 corpus, available within Python package [28] and listed in S1 Dataset.

**Fig 4:**
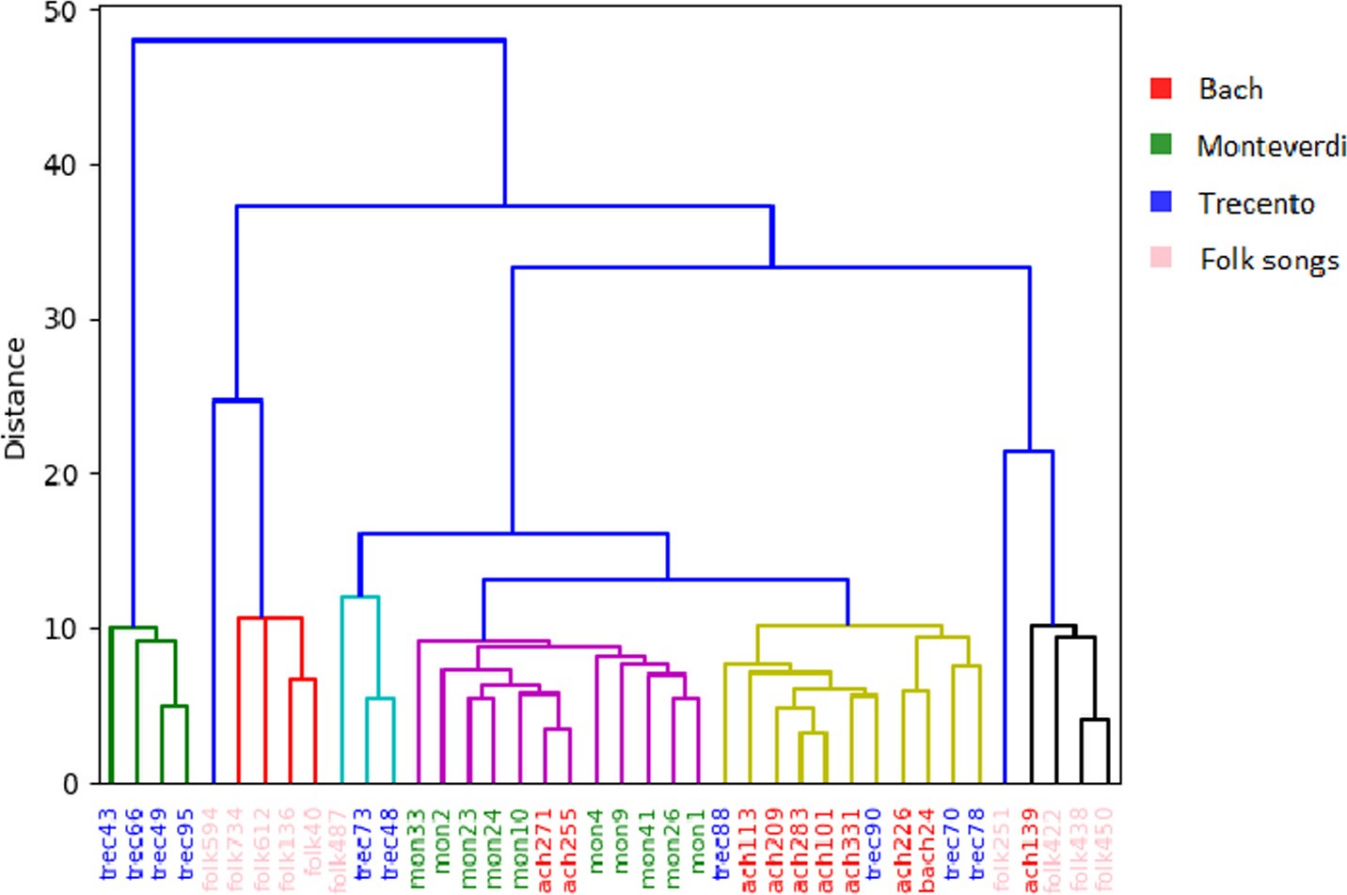
Results of hierarchical clustering of 40 compositions from 4 classes (3 classical composes and 1 music genre). Original classification of compositions is marked with text color of composition identifiers (red, green, blue and pink). Coloring of dendrogram branches which correspond to output groups is unrelated to class coloring. Groups in the dendrogram are assigned random colors, other than blue.

**Fig 5:**
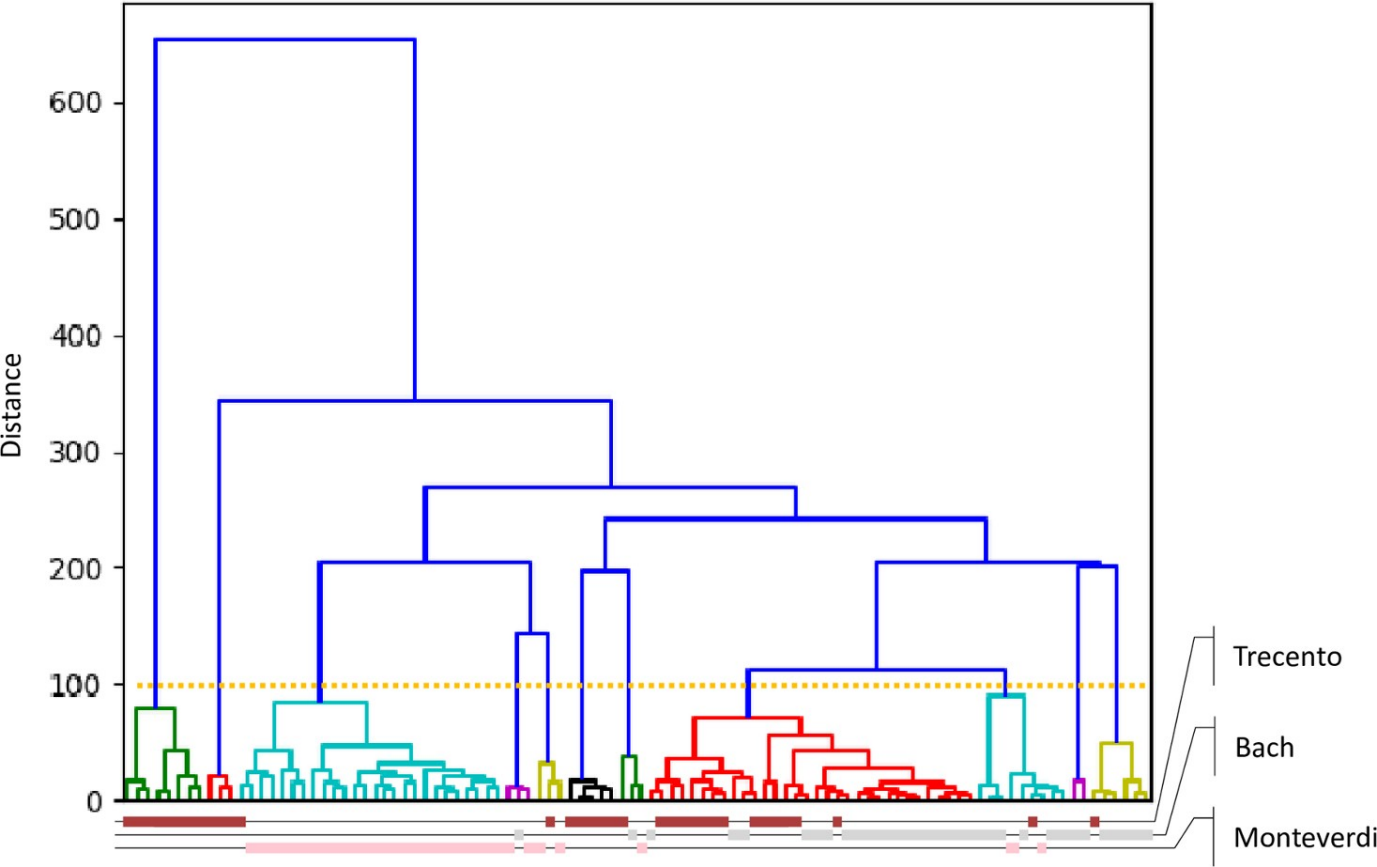
Results of hierarchical clustering of 90 classical pieces from 3 input classes. Graph layout is as in Fig 4. Additionally, grouping threshold is marked with golden dotted horizontal line.

In Figs 4 and 5 we present dendrograms of hierarchical clustering that we conducted on random and balanced subsets of our musical corpora in order to visualize grouping results. The way works have got grouped into branches does differ somewhat from their original attribution to “Bach”, “Monteverdi”, “Trecento” and “Folk songs” classes. Despite this we can notice that generally compositions from the same input class tend to obtain highest similarity score. We also can adjust the cutoff threshold that divides the collection into groups in which one of the initial classes dominates.

Grouping results turn out to be very instructive. For example, Monteverdi’s works are particularly likely to be grouped together. If a composition turns out to be similar to a piece from a different input class, it is rather a Bach’s work than a melody from trecento period—despite it is rather Monteverdi, not Bach, that shares Italian origin with the trecento composers. This result is consistent with our knowledge of music history. Claudio Monteverdi opposed his music to compositions from the Renaissance as a pioneer of a new trend in classical baroque music called *stile moderne*, whereas works of the trecento period are considered as the beginnings of Renaissance polyphony. Johann Sebastian Bach wrote over a century later than Claudio Monteverdi, but both of them represent baroque music. Among Bach’s musical inspirations there are not only chorale and German organ schools but also Italian instrumental and vocal-instrumental music. Another musicological explanation of this result is the type of compositions. Monteverdi and Bach created mainly sacred music, while the music of the Renaissance and trecento period is primarily secular works.

### 4.3 Correspondence with input classes

**Fig 6:**
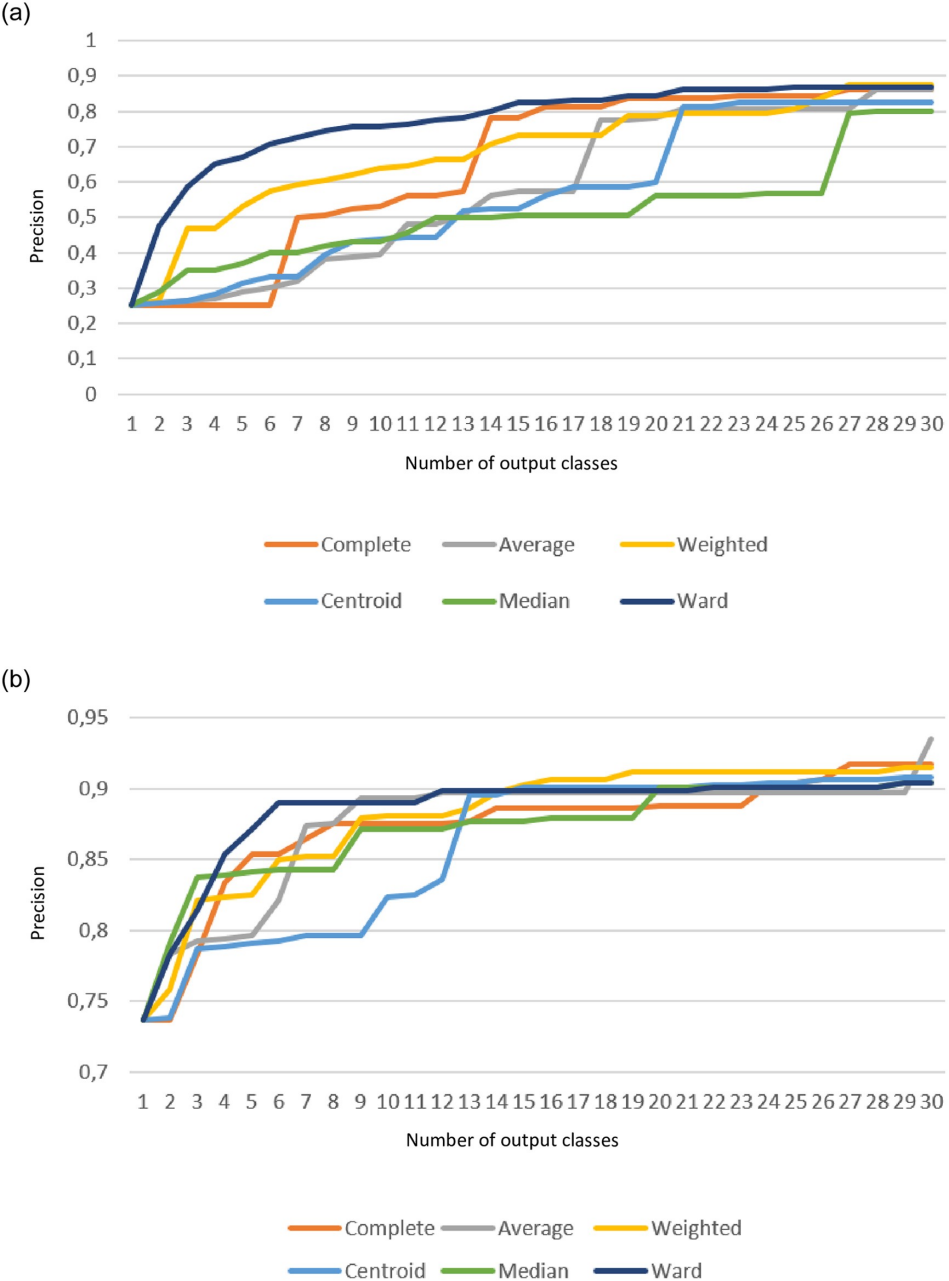
Grouping accuracy w.r.t. the number of output groups. (a) balanced collection of random samples of music pieces from classical and folk music compositions collections. (b) the entire unbalanced collection of classical works.

Fig 6 presents grouping precision as defined in (13) for our algorithm, w.r.t. the number of desired final groups. We carried out two experiments: one for a balanced set of 160 random pieces from all four input classes (three classical and one folk), and another one for unbalanced set of classical music only. In each experiment we have tested six standard linkage methods, i.e. variants of calculation of distance *z* between two already existing groups of pieces, *H*_1_ = {*M*_11_, *M*_12_, …} and *H*_2_ = {*M*_21_, *M*_22_, …} that are considered to be joined. These are:

1. complete distance—the maximum pairwise distance between pieces from each group, *z*(*H*_1_, *H*_2_) = max_*i*,*j*_ *ζ*(*M*_1*i*_, *M*_2*j*_)^−1^;
2. average distance between pieces being joined;
3. weighed average, with weighs calculated in previous grouping steps, recursively;
4. distance between centroids of *H*_1_ and *H*_2_;
5. the median of distance between pieces being joined;
6. Ward’s method, which calculates increase of variance for the joined group w.r.t. the initial state.

In our experiments, different grouping methods performed better. Grouping based on Ward’s linkage provided high precision (0.65) in case of only four output groups of compositions in the first experiment (cf. Fig 6a). Every linkage method hit a score of about 0.8 when we divided collection in more than 20 groups. Second experiment (cf. Fig 6b) for only classical compositions achieved higher precision, and we can see that median linkage method was a big success for three output groups (0.8376), as well as Ward’s linkage method for six groups (0.8899).

### 4.4 Discussion

To assess the value of our algorithm, we return to the analysis of related works on the classification of music compositions. Experiments by Herremans *et al*. who classified pieces of three composers: Bach, Haydn and Beethoven, reached accuracy from 80 to 86% and AUC (Area Under ROC Curve) beetween 79% and 93% [23]. That approach makes it possible to draw some statistical musical conclusions, for example, how often composers used particular intervals. Our classification model does not provide similar accuracy because our design goal was different. We wanted to provide primarily a grouping method whose advantage would be to detect musical similarities between works, based soundly on music theory. We consider this to have been achieved, cf. in particular occasional similarity of Monteverdi’s and Bach’s works discussed in Sec. 4.2. Previous studies on the similarity of compositions or classification were based on factors such as frequency of intervals [23] or rank-frequency distribution [22], which are not commonly used in musical analysis.

Agglomerative clustering used by us allows to set an arbitrary number of output groups of works, while typical classification task such as in [23] is just a special case when numbers of input classes and output groups are equal. Our results for more output groups than input classes are similar to earlier studies [29]. Whereas neural classification models provide superb accuracy (98% success rate in [22] or 94–97% precision in [30]), they are opaque and do not improve musicological understanding of analyzed music material.

## 5 Conclusion

The analogy between text documents and musical texts is evident. Reading music by musicians is similar to reading natural language. Such a comparison seemingly opens the way to musical analysis based straight on existing natural language approaches and tools like Apache Mahout [31]. However, a closer look reveals that the similarity limits itself to a vocabulary of terms, while musical syntax and “phraseology” are all different from linguistic. Moreover, with strict principles of music theory and harmony, its meaning may actually depend on the experience of the performer and listener. In addition, some music expressions can only be understood in the context of the entire music work. And last but not the least, polyphony in music has no corresponding phenomenon in literature whatsoever.

We have presented an approach to analyse and compare musical compositions based on musical content and the basic melodic elements corresponding to words in natural language. The advantages of this approach, despite its lower accuracy than other methods [22, 23], are clarity of reasoning and potentially high musicological value. One can extract easily form our grouping results the factors determining similarity of works put together. The result of our method is not only the partition of the collection of compositions but also the explanation why the melodies have been considered similar. A computer tool based on our solution could significantly improve research of musicologists. Our approach could also be adapted to drive music anti-plagiarism systems. Existing computer tools for music genres classification which are based on machine learning and neural networks can be improved by our musical content analysis [30]. It can also support music recommendation systems based on historical data of users preferences.

The presented method brings the following original contributions into the field:

- the motive is defined as a set of realizations, which addresses flexibility concept in the general popular definition of a motive; yet our approach is more comprehensible than the structural one involving graph theory;
- a weighing method is proposed, with weights reflecting composition practice;
- given the set-theoretic description of musical pieces, a combinatorial formula of calculating their similarity is proposed.

The apparatus presented here can be developed further in three areas: i) motives recognition, ii) computing motives and compositions similarity, iii) choosing a classification method.

In motives recognition, we will consider harmony. Defining the harmonic function and finding repetition of that function sequences can be the first step in motives recognition algorithm. We think that it could improve the quality of relevant motives found. The second improvement in motives recognition is considering the accents in notes sequences: both weak and strong parts of the bar as well as additional articulation markings.

Groups of motives realisations sometimes turn out too big when we want to account for as many composing techniques as possible, also by considering *n*-grams of many lengths. Further work should lead to decrease of the number of motive realisations through considering only the most important realisations and through limitation of considered composing techniques by musicological specialists, or by valuing their impact on motive creation.

The similarity of musical pieces in our research depends on the motives contained. We believe that research on extending the definition to include *n*-grams of motives would be valuable. Harmonic functions can be an additional factor in similarity value. It is also significant what function starts and ends a song, which function sequences form cadences or what additional notes occur in chords. This is particularly important in jazz music, which is often improvised and written only in the form of chords [32].

Most of the future work that we suggest should be supported by direct assistance of musicologists. An approach to computer musical analysis based on musical content and analogous to musicological analysis can be a common denominator for musicologists and computer scientists whose mutual understanding is still difficult [33, 34].

## Supporting information

S1 Dataset File names of classical music pieces from classes “Bach”, “Monteverdi” and “Trecento”. (TXT)

S2 Dataset File names of folk music pieces. (TXT)
